# Females and Their Diseases

**Published:** 1848

**Authors:** 


### Part 3.—Bibliographical Notices.

ARTICLE I.

*Females and their Diseases, a series of Letters to his Class.* By CHAS. D. MEIGS, M.D., Prof. of Midwifery and Diseases of Women and Children in the Jefferson Medical College at Philadelphia; Member of the American Medical Association, &c., &c. pp. 670. Phila.: Lea & Blanchard. 1848.

Here is a "new book," sure enough, as the author calls it; not that the teachings are all new, but new in manner, style, and the rapidity of its production.

Dr. Meigs has certainly attained one object which he set out to accomplish: we mean, to write in a familiar style.

Some may think he has "sacrificed the dignity" in this; but certatnly it is better to dispense with the stiff, prosey, formal rules of writing, to make a book readable, than to say many good things and have them printed for the coating of dust on the shelf.

Dr. Meigs may have introduced too much familiar sick-room conversation into his letters, and his style may be rather quaint for the best, but certainly, his great reputation, the change in the book from the usual manner of writing, and the intrinsic merits with which the work abounds, will give it a wide spread circulation, and a very general perusal.

Some points that are new in the body of the work we may take occasion hereafter to refer to, but have room now only to give the following as a specimen of the sick-room conversations. After an extensive interrogation, which we omit, the diologue goes on as follows:

"I mean that, as you are pregnant some five weeks, the womb is much larger than it was before you were married; and it is now turned over backwards;—in fact, it is topsy turvy. The pressure of the top of your womb against the lower part of the bowel and the back bone, gives you pain in that situation; while the bottom, or rather the point of the womb, is pressed with force against the bladder of urine, vexing and paining it, and stopping the course of the water, which can only escape drop by drop, while your bladder becomes continually fuller and fuller. It is very full now."

"Why, what in the world is to be done?"

"You are to allow me to remedy the difficulty."

"How?"

"By replacing the womb in its natural position."

"But how can you do that?"

"With my hand."

"I can't think of such a thing."

"Very well, madam, I shall have to bid you a good afternoon, for I can't think of anything else. In fact, there's nothing else to be done for you."

"Why, I'd rather die."

"As you please, madam; you are the mistress; ce que femme veut, Dieu le veut; but I hope you will permit me to say it would be very silly of you to die, for want of the power to make water, when there's a physician at hand, can put you so easily in the way to do it easily."

And she resorted to the ladies' resort—tears. After she had comforted herself a little in this way, and had come to her calmer reason again—

"Will it hurt me, sir?"

"Oh no, not a bit."

"What am I to do, then, if I must?"

"You are to lie on your left side, in bed. Covered up head and ears, with the bed-clothes. You are to draw up your knees very high, and I will cure you in a moment."

I washed my hands; I always do that first. I dipped the index finger of the right hand in oil. With its point I felt the gravid bas-fond of the womb looking downwards and forwards in the pelvis, while the os tincæ was cocked up against the urethra above the arch. Look again at the wood-cut.

"Don't bear down now, child! don't resist the pressure of my hand! I shall not hurt you at all, not in the least!" and so by a gentle, steady, augmented pressure against the bas-fond, pressing it upwards and backwards, I followed the ascending fundus until it suddenly escaped above the promontory, upon which the os uteri looked downwards again, and the organ was reposited."

"Now, how do you feel?"

"Oh, greatly relieved."

"Set up, and try to make water. I shall retire from your chamber. Call me when you are done. Is there a wash stand in the next chamber?"

In a few minutes she called me back; thankfully telling me she had made a large quantity of water, and was quite well again.

"Is not this, bad as it is, better than dying?"

"Oh yes, sir, thank you!" &c., &c.

